# The Stone Within: A Rare Case of an Incarcerated Inguinal Hernia With a Giant Fecaloma

**DOI:** 10.7759/cureus.82279

**Published:** 2025-04-15

**Authors:** Filipe Ramalho de Almeida, Joana Frazão, Rita Pera, João Guimarães

**Affiliations:** 1 General Surgery, Hospital Prof. Doutor Fernando Fonseca, Amadora, PRT; 2 General Surgery, Hospital Trofa Saúde Amadora, Amadora, PRT; 3 General Surgery, Unidade Local de Saúde do Médio Tejo, Tomar, PRT

**Keywords:** fecaloma, incarcerated inguinal hernia, inguinal-scrotal hernia, obstipation, sigmoid colon

## Abstract

Chronic constipation can lead to severe complications, including the formation of fecalomas, which may result in intestinal obstruction. Inguinal hernias, among the most prevalent abdominal wall hernias, can become incarcerated, leading to obstruction and ischemia. However, the occurrence of an incarcerated inguinal hernia containing a fecaloma is exceedingly rare. We present a case of a 72-year-old male who presented with a progressively enlarging left inguinal mass, urinary incontinence, and chronic diarrhea. Imaging studies revealed a significantly large left inguinoscrotal hernia containing the sigmoid colon impacted by a 4-kg fecaloma. Attempts at conservative disimpaction were unsuccessful, necessitating surgical intervention. The patient underwent a sigmoid colectomy with side-to-end colorectal anastomosis, hernia repair with prosthetic reinforcement, and a diverting ileostomy. This case highlights the importance of tailored surgical approaches in managing uncommon complications stemming from chronic constipation.

## Introduction

Chronic constipation is a common condition that, in extreme cases, can lead to fecal impaction and the formation of fecalomas, defined as masses of hardened, desiccated stools [1,2]. Fecalomas are most frequently found in the sigmoid colon and rectum, with their incidence increasing with age, significantly impacting patients' quality of life [1,3].

An inguinal hernia is the protrusion of any peritoneum-covered viscus through the inguinal canal. In approximately 10% of cases, incarceration of the hernial sac may occur, potentially leading to intestinal obstruction, strangulation, and ischemia [4,5].

Fecaloma-related hernia incarceration is likely underreported in the literature. We present an anecdotal case of an incarcerated inguinal hernia containing the sigmoid colon with a fecaloma.

## Case presentation

A 72-year-old man, with a history of ischemic heart disease, arterial hypertension, and hyperuricemia, presented to the emergency department with a 15-day history of urinary incontinence and mucous-containing diarrheal stools.. He also reported a loss of appetite over several months. Additionally, he mentioned a progressively growing left inguinal mass following inguinal hernia surgery around six years ago.

**Figure 1:**
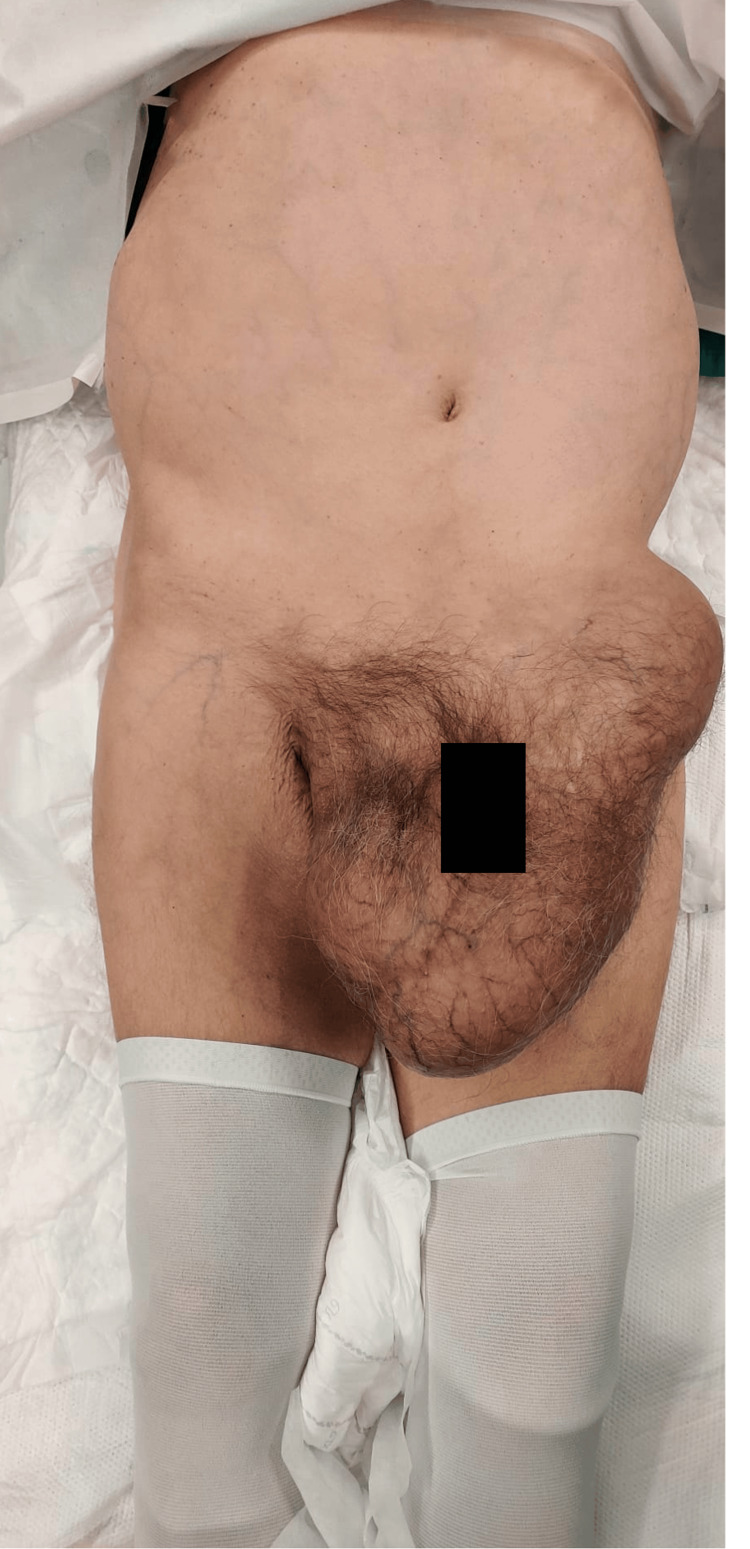
Patient presentation.

He displayed poor hygiene care, and on physical examination, there was abdominal distension with a large, indurated, painless mass extending from the left flank to the scrotal sac (Figure 1). Analytically, notable findings included mild leukocytosis with neutrophilia, a mild elevation of C-reactive protein, acute kidney injury, and hypokalemia (Table 1).

**Table 1:** Laboratory findings. CRP, C-reactive protein; MCH, mean corpuscular hemoglobin; MCHC, mean corpuscular hemoglobin concentration; MCV, mean corpuscular volume; RDW (CV), red cell distribution width (coefficient of variation)

Parameter	Patient values	Reference range
Hemoglobin	14.8	13.0–17.0 g/dL
Erythrocytes	4.74	4.50–5.50 x10¹²/L
Hematocrit	42.7	40.0–50.0 %
MCV	90.1	78.0–98.0 fL
MCH	31.2	27.0–32.0 pg
MCHC	34.7	(32.0–36.0)
RDW (CV)	13.2	<15.0 %
Leukocytes	11.4	4.0–10.0x10⁹/L
Neutrophils	81.2 (9.3)	% (1.8–6.9x10⁹/L)
Eosinophils	0.7 (0.1)	% (< 0.6x10⁹/L)
Basophils	0.3 (0.0)	% (< 0.1x10⁹/L)
Lymphocytes	12.2 (1.4)	% (1.2–3.3x10⁹/L)
Monocytes	5.6 (0.6)	% (0.2–1.0x10⁹/L)
Platelets	246	150–410x10⁹/L
Sodium	133.1	136.0–145.0 mmol/L
Potassium	3.02	3.50–5.10 mmol/L
Chloride	93.7	98.0–107.0 mmol/L
Creatinine	2.09	0.70–1.20 mg/dL
Urea	149.6	< 50.0 mg/dL
Glomerular filtration rate	33.00	mL/min/1.73m²
CRP	2.62	< 5.0 mg/dL

**Figure 2:**
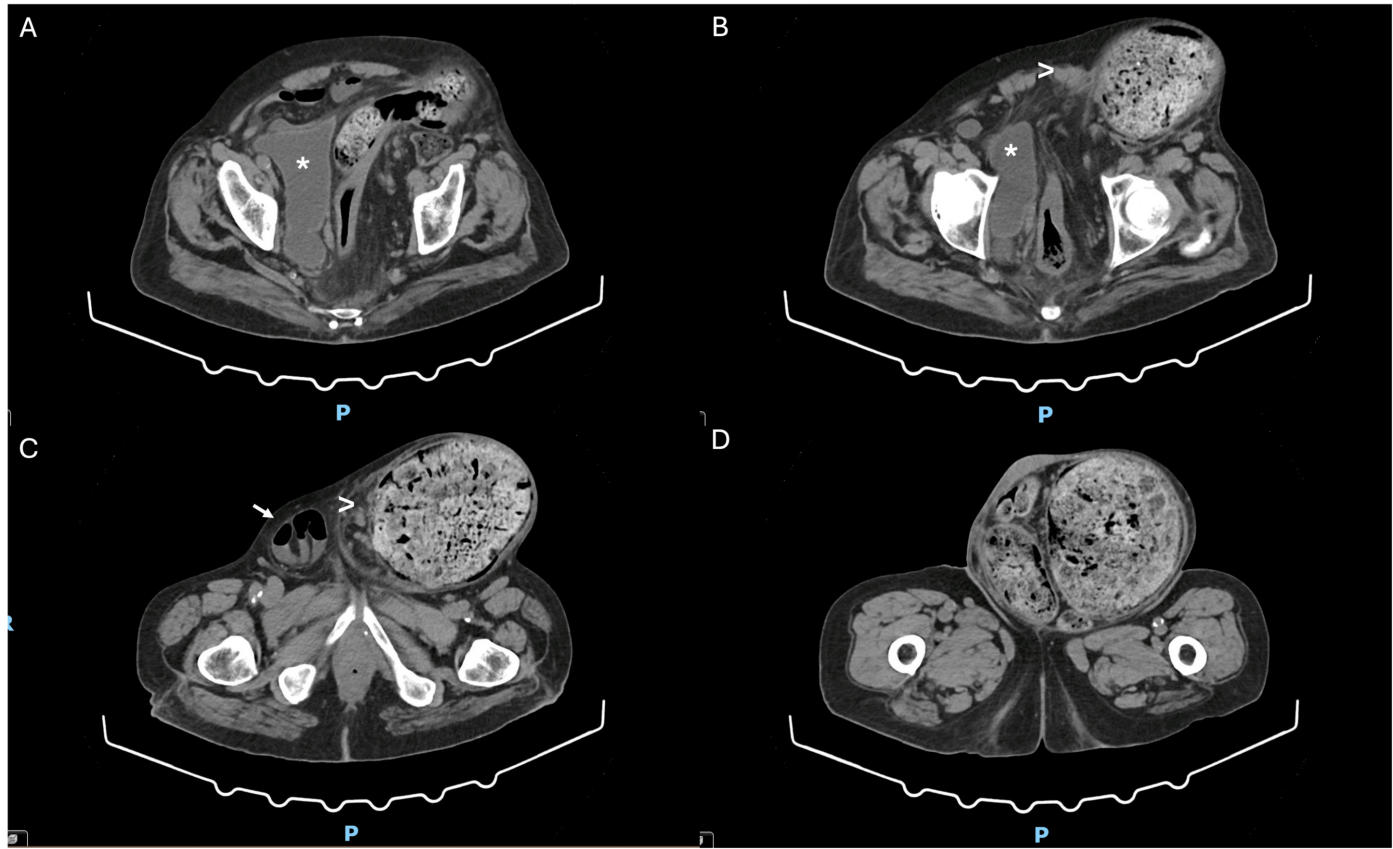
CT scan. CT scan images, from cranial to caudal (A-D), show a voluminous (maximum diameter of 15 cm) inguinoscrotal hernia containing the sigmoid colon dilated and filled with a giant fecaloma. Mild mesenteric fat densification (arrowhead) is also noted inside the hernial sac and its pedicle. On the right inguinal canal, there is an additional hernia containing an ileal loop (arrow). The alterations cause the deformation and displacement of the bladder (*) to the right.

A computed tomography scan (Figure 2) revealed a dilatation of the colon originating from a huge left inguinoscrotal hernia, containing the sigmoid colon, with a maximum diameter of approximately 15 cm, which was filled with a massive, “fossilized” fecaloma with an estimated volume of around 4 kg. A slight densification of mesenteric fat within the hernia and its pedicle was also noted. There was also an additional rectal fecaloma with distention of the rectum.

Attempts at fecal disimpaction using enemas were unsuccessful. The patient was admitted with a diagnosis of incarcerated inguinal hernia containing an exuberant fecaloma within the herniated colon, and surgical intervention was decided to resolve the condition.

**Figure 3:**
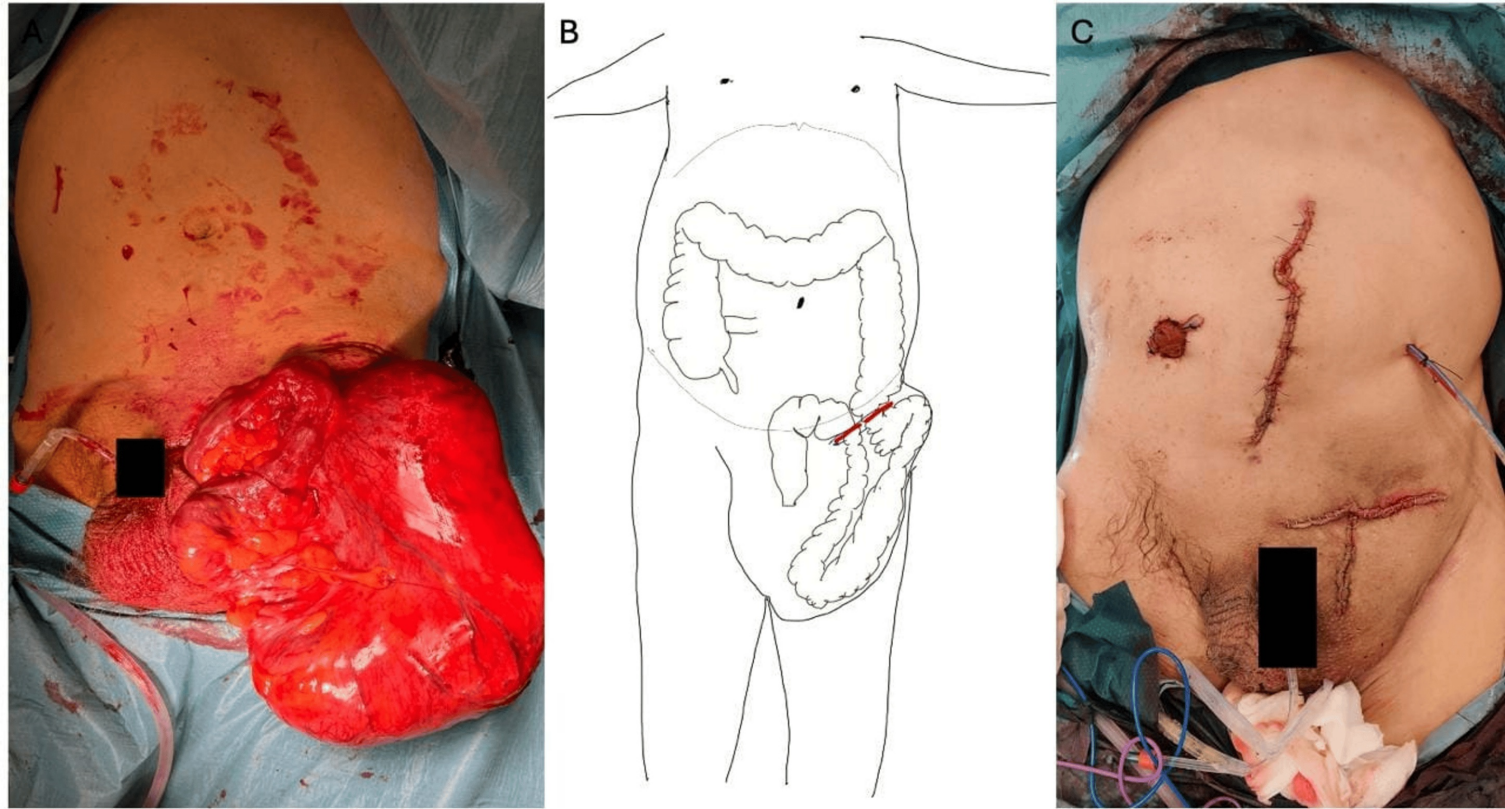
Intra-operative images. A. Hernial sac after dissection and liberation from the scrotal sac. B. Scheme of the section area (red lines) (original schematic representation). C. Patient after surgery.

**Figure 4:**
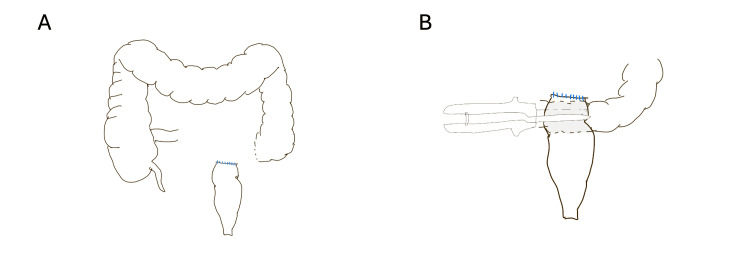
Anastomosis scheme. A. Colon and rectum after sigmoid colectomy. B. Colorectal side-to-end anastomosis on the posterior face of the rectal stump using a GIA™ stapler (60-mm purple cartridge) (original schematic representation).

The patient underwent a left inguinotomy, which was extended with a vertical incision to the scrotum due to difficulty in completing the hernial sac dissection (Figure 3A). The hernial sac was opened after its dissection with identification and preservation of the cord elements, and it was verified that it contained the sigmoid colon with a large fecaloma (15x25 cm). It was decided to perform a segmental resection of the sigmoid colon using GIA™ staplers, needing three 60-mm purple cartridges for the distal end due to the dilatation of the colon and one for the proximal end (Figure 3B). After that, complete destruction of the inguinal floor was observed, with a hernial defect of approximately 10 cm. A plug of prosthesis material was also identified near the pubis and Cooper's ligament, which was removed. A midline laparotomy was then performed to complete the anastomosis. Due to colon distention and the presence of fecalomas, an appendectomy was performed for colonic lavage, and colonic milking was done. A “functional” side-to-end colorectal anastomosis (Figure 4) was performed due to the stumps' diameter difference using a GIA™ 60-mm purple cartridge. The peritoneum covering the hernial defect was closed via abdominal access. The hernial defect was repaired via an anterior approach by creating a two-layer inguinal floor using the hernial sac remnants. A flat prosthesis was applied and secured using 2/0 Prolene® sutures to Cooper's and the inguinal ligaments, reinforcing the hernial repair. A diverting ileostomy was created, and a suction drain was left near the colorectal anastomosis (Figure 3C). The patient was put on empiric ciprofloxacin and metronidazole for seven days.

**Figure 5:**
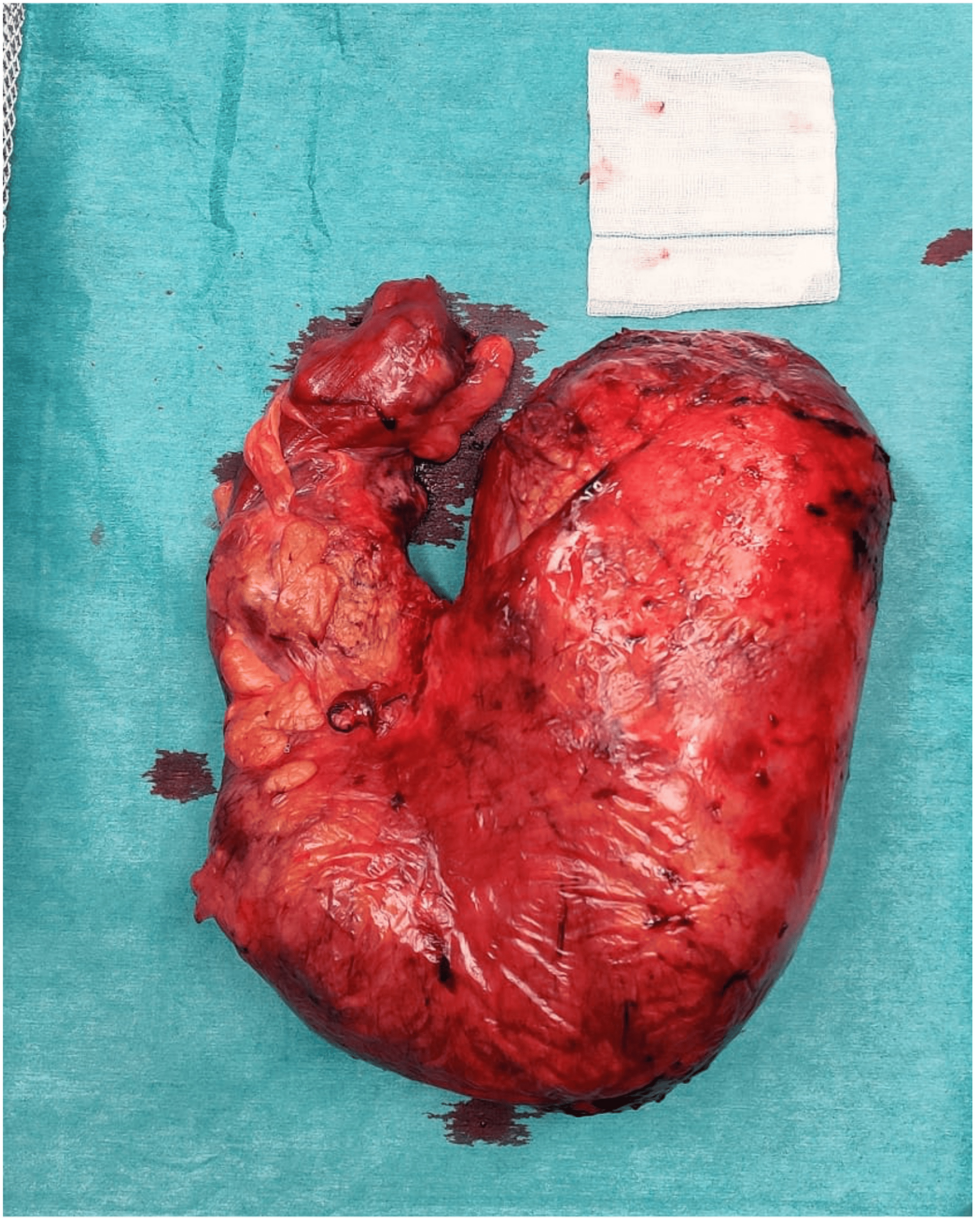
Surgical specimen.

The histopathological examination of the surgical specimen (Figure 5) documented a colonic segment measuring 77 cm in length, with a circumferential perimeter ranging from 4.5 cm to 14 cm. There was a decrease in wall thickness and a reduction in the natural folding pattern, which was more pronounced from the narrower to the more dilated margin. Additionally, areas of mucosal erosion, marked submucosal edema, and steatonecrosis of the pericolonic fat were observed.

In the postoperative period, the patient, who was reliant on adult diapers, was catheterized to minimize the risk of surgical wound contamination. Despite this precaution, the patient developed a scrotal surgical wound infection, which was effectively managed with wound care. Additionally, the catheterization led to a urinary tract infection caused by Escherichia coli, which was treated with a course of fosfomycin. The patient was discharged on the seventh postoperative day, awaiting placement for convalescent care. Currently, one year after surgery, he is awaiting ileostomy closure.

## Discussion

Fecalomas represent the extreme spectrum of intestinal constipation and impaction, predominantly occurring in the rectum (70%) and sigmoid colon (20%). The risk factors associated with fecaloma formation closely resemble those linked to chronic constipation, particularly those related to colonic motility, adequate dietary fiber intake, and hydration. Consequently, fecalomas are more prevalent in elderly patients and individuals with neuropsychiatric disorders. The etiology of fecalomas can be categorized into three primary groups: chronic constipation (which encompasses metabolic factors such as hypothyroidism, diabetes mellitus, uremia, porphyria, and hypercalcemia; dietary factors related to insufficient fluid and fiber intake; medications including opiates, antipsychotics, iron preparations, and calcium channel blockers; neurogenic causes such as spinal cord injury, multiple sclerosis, and Parkinson's disease; and psychiatric illnesses), anatomical anorectal abnormalities (such as megarectum due to conditions like Chagas disease and Hirschsprung's disease, and anorectal stenosis from strictures or neoplasms), and functional anorectal abnormalities (including increased rectal compliance due to irritation or endometriosis, abnormal rectal sensation as seen in Crohn's disease, and pelvic floor dysfunction such as rectocele and enterocele or non-relaxing puborectalis) [1,3].

Patients with fecaloma may exhibit symptoms such as abdominal pain and distension, nausea, vomiting, and anorexia. In severe cases, complications such as intestinal obstruction and perforation may arise. Diagnosis typically relies on clinical history, physical examination (including digital rectal examination), and imaging studies such as abdominal X-ray or CT [1,3].

Most fecalomas are managed conservatively through oral laxatives, enemas, and manual disimpaction. Surgical intervention is indicated when conservative measures fail or if complications arise, such as volvulus or intestinal perforation [1-3].

Inguinal hernias are the most common type, accounting for 73% of cases, primarily due to the inherent muscular weakness of the inguinal region. The hernia sac can contain various abdominal structures, with the ileum being the most frequently involved. Incarcerated hernias are irreducible but still maintain blood supply, while strangulated hernias exhibit compromised blood flow, leading to ischemia. Approximately 10% of inguinal hernias become incarcerated, potentially resulting in strangulation, bowel obstruction, or ischemia [4,5]. The occurrence of herniation involving a portion of the intestinal tract containing a fecaloma is rare, with only six cases documented in the literature [2,6-10].

The surgical management of an incarcerated inguinal hernia containing a fecaloma involves reducing the hernia contents and repairing the abdominal wall. In some instances, a sigmoid colectomy may be necessary to remove both the fecaloma and the affected segment of the colon. The hernioplasty techniques utilized in hernia repair may vary, but the overarching goal remains to reinforce the inguinal region and prevent recurrence [2].

In the case presented, a sigmoid resection was performed due to the inability to reduce the herniated sigmoid colon and the unsuccessful attempts to mobilize the fecaloma through milking and manual transanal extraction. Although a colotomy for manual removal of the fecaloma was considered, this option raised concerns about fecal contamination of the inguinal region, which could compromise the use of prosthetic mesh for hernia repair due to increased infection risk. Given the significant caliber discrepancy between the rectal and colonic stumps, a side-to-end colorectal anastomosis was performed, as illustrated in Figure 4. Additionally, the redundant hernia sac was used to reinforce the inguinal canal floor with a double-layer closure, effectively separating the anastomosis from the prosthesis and reducing the risk of infection in the event of anastomotic dehiscence. This technique also contributed to strengthening the inguinal defect repair. Ultimately, a diverting ileostomy was constructed to minimize the risk of hernia repair failure in the event of anastomotic dehiscence. An alternative strategy would have been Hartmann’s procedure; however, intraoperative findings supported the feasibility and safety of a primary anastomosis. Furthermore, given that our institution has a significantly higher rate of ileostomy reversals compared to Hartmann’s reversals, the decision favored this approach. These combined strategies aimed to reduce the risk of surgical site infection while enabling the safe use of prosthetic mesh for hernia repair.

The patient developed a scrotal surgical wound infection and a urinary tract infection, both managed successfully, but these complications underscore the importance of preventive strategies such as tailored antibiotic prophylaxis, vigilant wound care, and close postoperative monitoring. While scrotal drains or pressure dressings were not used due to intraoperative findings, their selective use may help prevent complications such as scrotal hematoma or wound infection. In particular, pressure dressings could offer added support to the surgical site and reduce contamination risk. These interventions should be considered case-by-case, depending on patient factors and surgical context.

## Conclusions

This case illustrates the complexity of an incarcerated inguinal hernia containing a fecaloma, necessitating a specialized surgical approach to manage both the intestinal obstruction and hernial repair. After conservative measures failed, a sigmoid resection with side-to-end colorectal anastomosis was performed, accompanied by a two-layer inguinal floor reconstruction and prosthetic mesh placement. A diverting ileostomy was created to reduce postoperative risks, particularly those related to anastomotic dehiscence. This case underscores the importance of considering fecaloma as a differential diagnosis in patients presenting with incarcerated inguinal hernias, especially in the context of chronic constipation. It also highlights the critical need for individualized surgical planning and a multidisciplinary approach in managing complex abdominal wall and colorectal pathology.
